# The association of dietary pattern with the risk of common chronic diseases in Shandong Province, China: a cross-sectional study

**DOI:** 10.3389/fpubh.2025.1629284

**Published:** 2025-08-26

**Authors:** Xi Wu, Jianwei Liu, Xinyue Li, Wanxin Zhang, Yong Yang, Jiazi Ma, Mao Cao, Mengjie Cheng, Guangjian Wu, Haidi Xiu, Zhongjun Du

**Affiliations:** ^1^Shandong Academy of Occupational Health and Occupational Medicine, Shandong First Medical University & Shandong Academy of Medical Sciences, Jinan, China; ^2^Shandong Center for Disease Control and Prevention, Jinan, China; ^3^School of Public Health Jilin University, Changchun, China; ^4^College of Traditional Chinese Medicine, Shandong University of Traditional Chinese Medicine, Jinan, China

**Keywords:** dietary pattern, common chronic diseases, factor analysis, Chinese adults, cross-sectional study

## Abstract

**Background:**

Chronic diseases have emerged as a significant public health challenge, impacting the well-being of the Chinese populace, despite scant research exploring the influence of dietary factors on these conditions. This article aimed to investigate the dietary patterns of adult residents in Shandong Province, China, and explore the relationship between these dietary patterns and common chronic diseases.

**Methods:**

We used data from the Total Diet Study of the Population of Shandong Province in China between 2015 and 2016. After further screening, a total of 2,828 adult residents with complete dietary and chronic disease prevalence information were included in this study. Food frequency questionnaires were used to ascertain dietary consumption. Dietary patterns were derived through factor analysis. Multivariate logistic regression models were employed to assess the associations between dietary patterns and the risk of common chronic diseases, while adjusting for potential confounders.

**Results:**

Three dietary patterns were identified: dietary pattern 1 (characterized by high intake of grains and tubers, vegetables, fruits, eggs, meat, nuts, and legumes); dietary pattern 2 (with high consumption of edible fungi and algae, legumes, snacks, aquatic products, and vegetables, but low in eggs); and dietary pattern 3 (high in dairy, beverages, and snacks). Notably, dietary pattern 2 was associated with a decreased risk of coronary heart disease, even after adjusting for potential confounders [odds ratio (OR) = 0.25, 95% confidence interval (CI) = 0.08-0.79, *P* < 0.05]. A higher incidence of dyslipidemia was significantly correlated with dietary pattern 3 (OR = 2.33, 95% CI = 1.13–4.78, *P* < 0.05).

**Conclusions:**

Our findings demonstrated that adherence to specific dietary patterns can influence the risk of dyslipidemia and coronary heart disease. Higher adherence to dietary pattern 3 was linked to a higher risk of dyslipidemia, while dietary pattern 2 helped reduce the risk of coronary heart disease.

## 1 Introduction

The rapid economic growth and aging population in China have led to a significant rise in chronic non-communicable diseases, which have become a major public health concern. Data indicates that conditions such as cardiovascular diseases, diabetes, and cancers are prevalent, with notable urban-rural disparities and a trend toward younger onset ages. From 12.33% in 2003 to 34.29% in 2018, the prevalence of chronic diseases among the Chinese population has risen annually due to a variety of variables, including lifestyle, environment, and population age distribution (1). According to reports, 88.5% of all deaths among Chinese citizens in 2019 were caused by chronic illnesses, with diabetes, cancer, cardiovascular disease, and chronic respiratory conditions accounting for 47.1%, 24.1%, 8.8%, and 2.5% of deaths, respectively (2). The rapid increase in morbidity and mortality from chronic diseases poses a heavy burden on society, with up to 100.2 million disability-adjusted life years due to cardiovascular disease in 2021 alone (3).

Numerous studies show that dietary interventions are crucial in preventing chronic disease (4–7). However, single nutrient or food studies do not accurately reflect the overall effects of dietary combinations because of the interactions and synergy between nutrients (8). Hence, as an alternative way of explaining dietary complexity, dietary patterns better reflect reality. Factor analysis is one of the most commonly used methods in the study of dietary patterns and is widely used to capture different dietary characteristics in a population, to assess the overall quality of a diet and to construct statistical models (9, 10). The high-protein pattern extracted by factor analysis was found to be in negative association with the risk of dyslipidemia by Guo et al. (11). A previous cross-sectional study linked the cautious pattern with a higher prevalence of hypertension and coronary heart disease (CHD) (12). A 10-year cohort study showed that participants with higher adherence to traditional Nordic and modern dietary patterns had a lower risk of haemorrhagic stroke, ischaemic stroke and diabetes (13).

Due to a combination of factors such as geographical location, socio-economic situation, cultural practices and eating habits, there are great differences in the dietary structure of different groups of people (14, 15). The rapid development of Shandong Province in recent decades has dramatically changed the lifestyles and dietary habits of the local population (16). In 2018, Shandong had the highest age-standardized mortality rate and diet-related mortality rate of ischemic heart disease in China, with 92.8 deaths per 100,000 people (16). A large cross-sectional study showed that the prevalence rates of hypertension, diabetes and hyperlipidemia in rural areas of Shandong Province were 29.36%, 15.03% and 5.68% respectively (17). However, despite the prevalence of various chronic diseases in Shandong Province, there is a relative paucity of in-depth research on the potential links between specific dietary patterns and common chronic diseases in the region Consequently, this study aimed to identify the primary dietary patterns among adult residents in Shandong Province through factor analysis, and subsequently investigate the potential associations between these patterns and prevalent chronic diseases.

## 2 Materials and methods

### 2.1 Study participants

The data for this study was obtained from the Total Diet Study of the Population of Shandong Province (18). The project used a multi-stage, stratified, whole-cluster random sampling approach to recruit participants. Six municipal survey sites in Yantai, Weifang, Jinan, Liaocheng, Tai'an, and Linyi were chosen as they reflect the typical dietary habits and consumption levels of Shandong Province's residents, as evidenced by the province's rich food culture and specific dietary patterns. Subsequently, one district and two counties, each with a medium economic status, were selected from within each city. Two street offices per urban district and four neighborhoods per street office were selected; two townships per county and three villages per township were selected. The number of households taken from each community/village is not < 30. Survey respondents were household members or residents ≥2 years of age who had lived in the survey site for more than 6 months.

**Figure 1:**
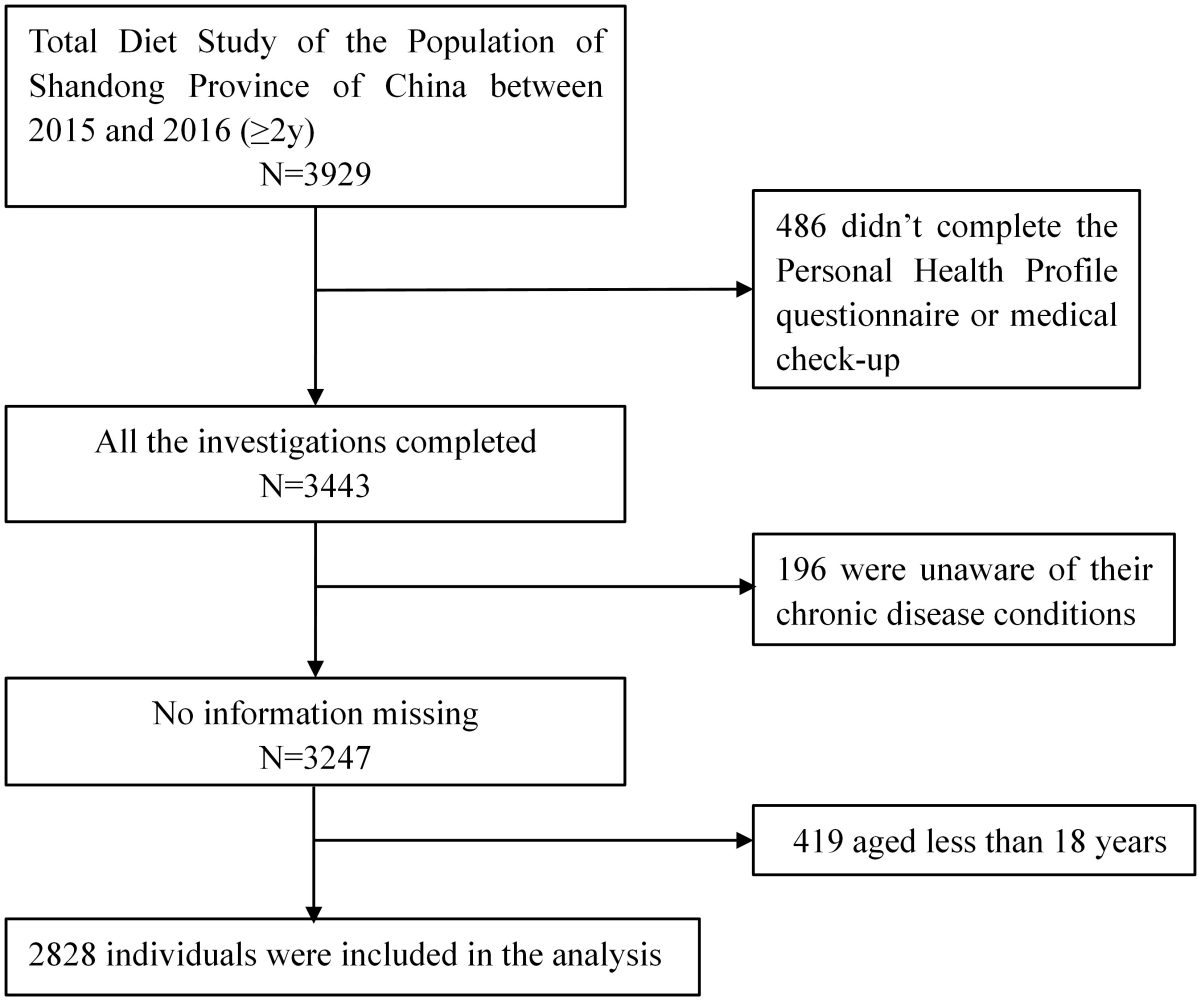
Flowchart of the participant selection process.

In the project, a total of 11,821 individuals from 3,667 households across Shandong Province were sampled, among whom 3,929 were investigated using the Food Frequency Questionnaire (FFQ). The project commenced on September 1, 2015, and concluded with a site survey at the end of March 31, 2016. The following were the inclusion criteria for this study: (1) participants who were 18 years of age or older; (2) completion of the Personal Health Profile questionnaire, FFQ, and a medical examination with no missing information. Ultimately, 2,828 participants were included in the final dataset. An informed consent was signed by each participant before the survey. The study was approved by the Shandong Academy of Occupational Health and Occupational Medicine in China, with the ethics number SDZFY-EC-H-2024-10. The screening flowchart was shown in Figure 1.

### 2.2 Dietary assessment

A standardized FFQ was used to measure dietary intake and gather data on participants' eating patterns throughout the previous 12 months. The 106 food items in the FFQ were categorized into 12 major groups, including grains and tubers, legumes, edible fungi and algae, fruits, vegetables, nuts, meat, eggs, aquatic products, dairy, snacks and beverages. Fractions from nutrient supplements, cooking oils and spices were not included in this study. The detailed food component categories were shown in Supplementary Table S1.

Participants were asked to fill in the number of intakes (daily, weekly, monthly, yearly, or never) and the weight of a single intake. The sum of the average daily intakes of all foods in each food group is the intake of that food group. Factor analysis was used to derive dietary patterns. The dietary sample data was first subjected to the Bartlett's test of sphericity and the Kaiser-Meyer-Olkin appropriateness test before factor analyses. The data was deemed appropriate for exploratory factor analysis if the Kaiser-Meyer-Olkin value was ≥0.5 and the P value for the Bartlett's test statistic was less than 0.05. Eigenvalues > 1 were used as criteria for extracting the common factors, which were then combined with the scree plot, cumulative contribution, and interpretability of the factors to determine the number of factors to retain, i.e., the main dietary patterns. Maximum variance rotation was used to obtain a more condensed factor structure. The intake of each food group and the associated factor loadings were used to compute factor scores for this dietary pattern. Each dietary pattern's factor scores were separated into tertiles, which rose from T1 to T3. The study participants' preference for the related food pattern increased with a higher score.

### 2.3 Measurement of anthropometry and other variables

Trained investigators used conventional procedures to perform medical examinations on the participants. A metal stadiometer with an accuracy of 0.1 cm was used to measure height. Weight was measured using electronic scales with an accuracy of 0.1 kg. Body mass index (BMI) was calculated by dividing weight in kilograms by height in meters squared (BMI = weight (kg)/(height (meters))^2^). Underweight (< 18.5 kg/m^2^), normal weight (18.5– < 25 kg/m^2^), overweight (25.0– < 30.0 kg/m^2^), and obese (≥30.0 kg/m^2^) were the BMI classifications (19). To test blood pressure, an electronic sphygmomanometer was used. A soft ruler was used to measure the participants' waist and hip circumferences to the closest 0.1 cm after they were instructed to change into light clothing. The measurement of waist circumference divided by hip circumference was used to compute the waist-to-hip ratio. For men and women, a healthy waist-to-hip ratio was < 0.9 and < 0.8, respectively; if not, central obesity was diagnosed (20).

Face-to-face enquiry surveys were conducted in households by trained and qualified enumerators, who collected comprehensive health-related information. Participants filled out a basic family profile registration form and a personal health profile questionnaire, which encompassed essential details about family members, demographic data (including gender, age, ethnicity, etc.), the current status of prevalent chronic diseases such as hypertension, diabetes, dyslipidaemia, stroke, and CHD, and family medical history.

### 2.4 Assessment of common chronic diseases

Adults with systolic blood pressure of 140 mmHg or higher and diastolic blood pressure of 90 mmHg or higher, tested three times on different days without the use of antihypertensive medications, were diagnosed with hypertension (21). Other diseases (diabetes mellitus, dyslipidaemia, stroke and coronary heart disease) were based on previous diagnosis in community/township hospitals and higher. The diagnosis of diabetes required a fasting blood glucose level of ≥7.00 mmol/L or self-reported diabetes (22). The diagnosis of dyslipidemia required meeting any of the following criteria: total cholesterol ≥6.22 mmol/L, or low-density lipoprotein cholesterol (LDL-C) ≥4.14 mmol/L, or high-density lipoprotein cholesterol < 1.04 mmol/L, or triglycerides ≥2.26 mmol/L, or a self-reported history of hyperlipidemia (23). In clinical practice, the diagnosis of stroke was mainly based on the symptoms of acute onset neurological deficits, imaging examinations, and biomarker measurements (24, 25). The diagnosis of coronary heart disease required a combination of typical angina pectoris symptoms, ischemic changes in electrocardiogram, elevated myocardial enzymes and coronary angiography (26).

### 2.5 Statistical analysis

Among this study, the basic features of patients in the various disease populations were reported after a descriptive analysis of each disease population. Means and standard deviations were used to characterize continuous variables, while rates and composition ratios were used to convey categorical variables. For measurement data, the *t*-test was used to compare differences, and for count data, the chi-square test was used for between-group comparisons.

The relationship between each dietary pattern and the risk of common chronic diseases was evaluated using logistic regression. In several models, the results were displayed as odds ratios (ORs) and 95% confidence intervals (CIs) in relation to the reference tertile, which was the lowest one. For this investigation, three models were created. Model 1 was a rudimentary model that did not account for variables. Age, gender, and BMI adjustments were made to Model 2. Based on Model 2, Model 3 was also modified to account for central obesity. SPSS 27.0 was used for all statistical analyses (IBM SPSS Inc., USA). In the study, a difference was deemed statistically significant if the bilateral *P* was < 0.05.

## 3 Results

### 3.1 Basic information of population

This study comprised 2,828 participants, ages 18 to 95, of which 1,361 (48.1%) were male and 1,467 (51.9%) were female. The characteristics of each participant are displayed in Table 1. Most participants (42.4%) fell within the age range of 18– 44. Regarding BMI, 46.5% of participants were overweight or obese, whereas 51.2% had a normal classification. Central obesity was present in 58% of the participants. 59.8% of the participants lived in cities, while the rest lived in rural areas.

**Table 1:** Demographic characteristics of participants.

**Variables**		**Number**	**Percent (%)**
Gender	Male	1,361	48.1
	Female	1,467	51.9
Age (years)	18–44	1,199	42.4
	45–59	933	33.0
	>60	696	24.6
BMI (kg/m^2^)	< 18.5	65	2.3
	18.5–25.0	1,448	51.2
	25.0–30.0	1,039	36.7
	≥30.0	276	9.8
Central obesity	No	1,187	42.0
	Yes	1,641	58.0
Region	City	1,690	59.8
	Rural	1,138	40.2

BMI, body mass index.

### 3.2 Characteristics of different affected populations

Table 2 displays the participants' characteristics based on their prevalence of common chronic conditions. The study subjects were grouped according to whether they were diseased or not. The prevalence of hypertension was 432, with a prevalence rate of 15.28%, and the difference between affected and unaffected residents was statistically significant for gender, BMI and central obesity (*P* < 0.05). The prevalence of diabetes mellitus was 125 with a prevalence rate of 4.42%, with differences in the distribution of gender and central obesity in both groups (*P* < 0.05). The prevalence of dyslipidaemia was 144 with a prevalence rate of 5.09%, it was significantly different in both groups in terms of gender and BMI (*P* < 0.05). The number of patients with CHD was 95, with a prevalence rate of 3.36%, and significant differences were observed with regard to gender, BMI and central obesity (*P* < 0.05).

**Table 2:** Characteristics of the study subjects according to common chronic diseases.

**Variables**	**Hypertension**			**Diabetes**			**Dyslipemia**			**Stroke**			**Coronary heart disease**		
	**No (n** = **2396)**	**Yes (n** = **432)**	**Significance** ^a^	**No (n** = **2703)**	**Yes (n** = **125)**	**Significance** ^a^	**No (n** = **2684)**	**Yes (n** = **144)**	**Significance** ^a^	**No (n** = **2786)**	**Yes (n** = **42)**	**Significance** ^a^	**No (n** = **2733)**	**Yes (n** = **95)**	**Significance** ^a^
**Age (%)**															
18–44	1,018 (42.5)	181 (41.9)	χ^2^ = 0.491	1,151 (42.6)	48 (38.4)	χ^2^ = 1.355	1,143 (42.6)	56 (38.9)	χ^2^ = 0.897	1,184 (42.5)	15 (35.7)	χ^2^ = 1.147	1,161 (42.5)	38 (40.0)	χ^2^ = 1.1
45–59	794 (33.1)	139 (32.2)	*P* = 0.782	886 (32.8)	47 (37.6)	*P* = 0.508	881 (32.8)	52 (36.1)	*P* = 0.638	919 (33.0)	14 (33.3)	*P* = 0.563	897 (32.8)	36 (37.9)	*P* = 0.577
≥60	584 (24.4)	112 (25.9)		666 (24.6)	30 (24.0)		660 (24.6)	36 (25.0)		683 (24.5)	13 (31.0)		675 (24.7)	21 (22.1)	
**Gender (%)**															
Male	1,237 (51.6)	124 (28.7)	χ^2^ = 77.045	1,327 (49.1)	34 (27.2)	χ^2^ = 22.939	1,325 (49.4)	36 (25.0)	χ^2^ = 32.503	1,344 (48.2)	17 (40.5)	χ^2^ = 0.999	1,329 (48.6)	32 (33.7)	χ^2^ = 8.212
Female	1,159 (48.4)	308 (71.3)	*P* < 0.001	1,376 (50.9)	91 (72.8)	*P* < 0.001	1,359 (50.6)	108 (75.0)	*P* < 0.001	1,442 (51.8)	25 (59.5)	*P* = 0.317	1,404 (51.4)	63 (66.3)	*P* = 0.004
**BMI (%)**															
< 18.5	59 (2.5)	6 (1.4)	χ^2^ = 21.983	61 (2.3)	4 (3.2)	χ^2^ = 4.692	63 (2.4)	2 (1.4)	χ^2^ = 22.813	65 (2.3)	0	χ^2^ = 6.261	61 (2.2)	4 (4.2)	χ^2^ = 8.095
18.5–25.0	1,267 (52.9)	181 (41.9)	*P* < 0.001	1,392 (51.5)	56 (44.8)	*P* = 0.184	1,400 (52.2)	48 (33.3)	*P* < 0.001	1,431 (51.4)	17 (40.5)	*P* = 0.082	1,408 (51.5)	40 (42.1)	*P* = 0.038
25.0–30.0	847 (35.4)	192 (44.4)		992 (36.7)	47 (37.6)		969 (36.1)	70 (48.6)		1,023 (36.7)	16 (38.1)		1,004 (36.7)	35 (36.8)	
≥30.0	223 (9.3)	53 (12.3)		258 (9.5)	18 (14.4)		252 (9.4)	24 (16.7)		267 (9.6)	9 (21.4)		260 (9.5)	16 (16.8)	
**Central obesity (%)**															
No	1,044 (43.6)	143 (33.1)	χ^2^ = 16.476	1,146 (42.4)	41 (32.8)	χ^2^ = 4.518	1,135 (42.3)	52 (36.1)	χ^2^ = 2.141	1,168 (41.9)	19 (45.2)	χ^2^ = 0.187	1,157 (42.3)	30 (31.6)	χ^2^ = 4.361
Yes	1,352 (56.4)	289 (66.9)	*P* < 0.001	1,557 (57.6)	84 (67.2)	*P* = 0.034	1,549 (57.7)	92 (63.9)	*P* = 0.143	1,618 (58.1)	23 (54.8)	*P* = 0.666	1,576 (57.7)	65 (68.4)	*P* = 0.037

BMI, body mass index. Categorical variables are presented as sum and percentages. ^a^*P* values for categorical variables (chi-square test).

### 3.3 Dietary patterns

We verified that the Kaiser–Meyer–Olkin index (0.756) and Bartlett's test (*P* < 0.001) supported the applicability of the factor analysis of the dietary data. After extracting the factors with feature values greater than 1, combined with the lithotripsy plot and the interpretability of the factors, three common factors were finally determined. The lithotripsy pattern is shown in Supplementary Figure S1. The eigenvalues of these three common factors were 2.76, 1.41 and 1.13 respectively, and the explanatory variances were 23.0, 11.7 and 9.4% respectively. These factors combined accounted for more than 44% of the variance in food intake observed. Table 3 displays the factor loadings for each of the three primary dietary patterns that this study's factor analysis revealed. Grains and tubers, vegetables, fruits, eggs, meat, nuts, and legumes were the staples of dietary pattern 1. Dietary pattern 2 was defined by a low intake of eggs and a high intake of edible fungi and algae, legumes, snacks, aquatic products, and vegetables. High consumption of dairy products, beverages, and snacks characterized dietary pattern 3.

**Table 3:** Factor-loading matrix for the three dietary patterns^a^.

**Food groups**	**Dietary pattern 1**	**Dietary pattern 2**	**Dietary pattern 3**
Grains and tubers	0.702	–	–
Vegetables	0.640	0.361	–
Fruits	0.634	**–**	–
Eggs	0.597	−0.329	–
Meat	0.504	–	–
Nuts	0.323	–	–
Edible fungi and algae	**–**	0.735	–
Legumes	0.314	0.528	–
Aquatic products	–	0.433	–
Dairy	–	–	0.708
Beverages	–	–	0.614
Snacks	–	0.516	0.586

^a^Absolute values < 0.3 were excluded for simplicity.

### 3.4 Associations between dietary patterns and common chronic diseases

The relationships between the three dietary patterns and CHD, stroke, diabetes, dyslipidemia, and hypertension are shown in Table 4. The chances of CHD were considerably lower for individuals in the highest tertile of dietary pattern 2 intake than for those in the lowest tertile (*R* = 0.19, 95%CI = 0.06–0.56, *P* < 0.05). When age, gender, BMI, and central obesity were taken into account, this connection persisted (OR = 0.25, 95%CI = 0.08–0.79, *P* < 0.05). After controlling for model 3 covariates, the incidence of dyslipidemia was higher for those in the highest tertile of dietary pattern 3 adherence than for those in the lowest adherence (OR = 2.33, 95% CI = 1.13–4.78, *P* < 0.05). Additionally, compared to the lowest tertile of dietary pattern 3, the highest tertile was adversely linked to hypertension. After controlling for other variables, however, this association between T3 and T1 vanished (model 1: OR = 0.61, 95% CI = 0.38–0.96, *P* < 0.05; model 2: OR = 0.93, 95% CI = 0.56–1.56, *P* = 0.79; model 3: OR = 0.94, 95% CI = 0.57–1.57, *P* = 0.82).

**Table 4:** Association between three dietary patterns and each chronic disease.

**Hypertension**		**Model 1** ^**a**^		**Model 2** ^**b**^		**Mode 3** ^**c**^	
**Tertile of dietary pattern scores**		**OR (95% CI)**	***P*** **-Trend**	**OR (95% CI)**	***P*** **-Trend**	**OR (95% CI)**	***P*** **-Trend**
Dietary pattern 1	T1 [Low]	1.00 (Ref.)		1.00 (Ref.)		1.00 (Ref.)	
	T2	0.83 (0.55–1.27)	0.391	1.08 (0.68–1.71)	0.750	1.06 (0.67–1.68)	0.813
	T3 [High]	0.94 (0.62–1.41)	0.752	1.09 (0.69–1.72)	0.708	1.06 (0.67–1.68)	0.793
Dietary pattern 2	T1 [Low]	1.00 (Ref.)		1.00 (Ref.)		1.00 (Ref.)	
	T2	0.70 (0.45–1.07)	0.099	0.91 (0.56–1.46)	0.687	0.91 (0.56–1.46)	0.686
	T3 [High]	0.66 (0.43–1.02)	0.063	1.03 (0.63–1.67)	0.917	1.03(0.63–1.67)	0.920
Dietary pattern 3	T1 [Low]	1.00 (Ref.)		1.00 (Ref.)		1.00 (Ref.)	
	T2	0.78 (0.51–1.21)	0.266	0.88 (0.55–1.43)	0.617	0.89 (0.55–1.45)	0.646
	T3 [High]	0.61 (0.38–0.96)^*^	0.031	0.93 (0.56–1.56)	0.794	0.94 (0.57–1.57)	0.816
**Diabetes**		**Model 1**		**Model 2**		**Mode 3**	
**Tertile of dietary pattern scores**		**OR (95% CI)**	***p*** **-Trend**	**OR (95% CI)**	***p*** **-Trend**	**OR (95% CI)**	***p*** **-Trend**
Dietary pattern 1	T1 [Low]	1.00 (Ref.)		1.00 (Ref.)		1.00 (Ref.)	
	T2	1.17 (0.54–2.58)	0.690	1.61 (0.71–3.69)	0.256	1.60(0.70–3.67)	0.263
	T3 [High]	1.00 (0.44–2.26)	1.000	1.14 (0.48–2.68)	0.766	1.13 (0.48–2.67)	0.775
Dietary pattern 2	T1 [Low]	1.00 (Ref.)		1.00 (Ref.)		1.00 (Ref.)	
	T2	0.84 (0.43–1.63)	0.603	1.09 (0.54–2.17)	0.818	1.05 (0.52–2.10)	0.893
	T3 [High]	0.53 (0.25–1.13)	0.101	0.76 (0.35–1.67)	0.500	0.72 (0.33–1.57)	0.403
Dietary pattern 3	T1 [Low]	1.00 (Ref.)		1.00 (Ref.)		1.00 (Ref.)	
	T2	1.24 (0.59–2.62)	0.575	1.39 (0.65–3.00)	0.400	1.39 (0.64–3.00)	0.401
	T3 [High]	0.76 (0.33–1.76)	0.525	1.09 (0.46–2.59)	0.841	1.09 (0.46–2.59)	0.841
**Dyslipemia**		**Model 1**		**Model 2**		**Mode 3**	
**Tertile of dietary pattern scores**		**OR (95% CI)**	***p*** **-Trend**	**OR (95% CI)**	***p*** **-Trend**	**OR (95% CI)**	***p*** **-Trend**
Dietary pattern 1	T1 [Low]	1.00 (Ref.)		1.00 (Ref.)		1.00 (Ref.)	
	T2	1.53 (0.72–3.22)	0.266	2.03 (0.93–4.45)	0.077	1.97 (0.89–4.32)	0.093
	T3 [High]	1.17 (0.54–2.58)	0.690	1.29 (0.56–2.95)	0.546	1.25 (0.55–2.86)	0.600
Dietary pattern 2	T1 [Low]	1.00 (Ref.)		1.00 (Ref.)		1.00 (Ref.)	
	T2	1.15 (0.55–2.40)	0.715	1.54 (0.72–3.31)	0.269	1.47 (0.68–3.18)	0.323
	T3 [High]	1.15 (0.55–2.41)	0.708	1.82 (0.84–3.94)	0.132	1.67 (0.77–3.64)	0.199
Dietary pattern 3	T1 [Low]	1.00 (Ref.)		1.00 (Ref.)		1.00 (Ref.)	
	T2	1.30 (0.63–2.66)	0.475	1.48 (0.71–3.08)	0.299	1.46 (0.70–3.04)	0.316
	T3 [High]	1.61 (0.81–3.21)	0.173	2.35 (1.14–4.83)^*^	0.020	2.33 (1.13–4.78)^*^	0.022
**Stroke**		**Model 1**		**Model 2**		**Mode 3**	
**Tertile of dietary pattern scores**		**OR (95% CI)**	***p*** **-Trend**	**OR (95% CI)**	***p*** **-Trend**	**OR (95% CI)**	***p*** **-Trend**
Dietary pattern 1	T1 [Low]	1.00 (Ref.)		1.00 (Ref.)		1.00 (Ref.)	
	T2	1.00 (0.32–3.13)	1.000	1.34 (0.41–4.35)	0.630	1.31 (0.40–4.26)	0.655
	T3 [High]	1.00 (0.32–3.13)	1.000	1.07 (0.33–3.47)	0.913	1.05 (0.32–3.41	0.937
Dietary pattern 2	T1 [Low]	1.00 (Ref.)		1.00 (Ref.)		1.00 (Ref.)	
	T2	1.00 (0.20–4.98)	0.997	1.45 (0.28–7.57)	0.658	1.51 (0.29–7.93)	0.629
	T3 [High]	1.00 (0.20–5.00)	1.000	1.85 (0.34–9.92)	0.475	1.98 (0.36–10.95)	0.432
Dietary pattern 3	T1 [Low]	1.00 (Ref.)		1.00 (Ref.)		1.00 (Ref.)	
	T2	1.61 (0.52–4.98)	0.408	1.70 (0.54–5.38)	0.367	1.69 (0.53–5.35)	0.375
	T3 [High]	0.40 (0.08–2.06)	0.271	0.55 (0.10–2.96)	0.488	0.55 (0.10–2.94)	0.485
Dietary pattern 1	T1 [Low]	1.00 (Ref.)		1.00 (Ref.)		1.00 (Ref.)	
	T2	0.90 (0.36–2.24)	0.816	1.23 (0.48–3.17)	0.665	1.20 (0.46–3.08)	0.710
	T3 [High]	0.80 (0.31–2.04)	0.634	0.97 (0.37–2.56)	0.947	0.94 (0.36–2.49)	0.900
Dietary pattern 2	T1 [Low]	1.00 (Ref.)		1.00 (Ref.)		1.00 (Ref.)	
	T2	0.53 (0.25–1.13)	0.099	0.72 (0.32–1.61)	0.429	0.69 (0.31–1.55)	0.371
	T3 [High]	0.19 (0.06–0.56)^*^	0.003	0.27 (0.09–0.85)^*^	0.024	0.25 (0.08–0.79)^*^	0.018
Dietary pattern 3	T1 [Low]	1.00 (Ref.)		1.00 (Ref.)		1.00 (Ref.)	
	T2	1.08 (0.49–2.41)	0.845	1.20 (0.53–2.75)	0.662	1.26 (0.55–2.88)	0.589
	T3 [High]	0.66 (0.27–1.63)	0.367	0.99 (0.39–2.53)	0.988	1.00 (0.39–2.55)	0.997

OR, odds ratio; 95% CI, 95% confidence interval; T, tertile; Ref., reference category. ^*^P < 0.05. The odds ratios across tertiles of dietary patterns were compared with that in the reference group (T1). ^a^Model 1: unadjusted model. ^b^Model 2: adjusted for age, gender, and body mass index. ^c^Model 3: additionally adjusted for central obesity.

## 4 Discussion

Three dietary patterns among adult residents of Shandong Province were extracted for this study using factor analysis. Dietary pattern 1 was characterized by high factor loadings for staple foods such as grains and tubers, as well as vegetables, fruits, eggs, meat, nuts, and legumes. Dietary pattern 2 was characterized by low egg consumption and high consumption of edible fungi and algae, legumes, snacks, aquatic products, and vegetables. Dietary pattern 3 tended to be highly loaded with dairy, beverage and snacks. Among these, a decreased risk of CHD was linked to greater adherence to dietary pattern 2. After accounting for a number of factors, a higher risk of dyslipidemia was linked to high adherence to dietary pattern 3. Conversely, there was no significant correlation found between dietary pattern 1 and CHD, stroke, diabetes, dyslipidemia, or hypertension.

In this study, we observed that participants who adhered to dietary pattern 2 experienced a significantly reduced risk of CHD, with a 75% lower incidence compared to those who did not follow this pattern. Numerous investigations into the connection between dietary patterns and CHD have been carried out (27–29). However, due to the frequent variation in the quantity and composition of food groups used in previous studies, as well as the significant differences in dietary patterns compared to dietary pattern 2, direct comparison of their results with ours is challenging. Dietary pattern 2's negative correlation with CHD risk may be due to a combination of the dietary components and the characteristics of the nutrients it contains. Edible fungi and algae in the FFQ included mushrooms, edible non-mushroom fungi (such as agaric and tremella), nori, and kelp. A recent systematic review found that eating mushrooms decreased blood triglyceride levels but did not correlate with the incidence of CHD (30). Meanwhile, agaricus bisporus, auricularia auricular, lentinula edodes and pleurotus ostreatus that we often consume are rich in glucan, which has cholesterol-lowering properties (31, 32). In addition, nori and kelp are also known to have lipid-lowering effects, as they contain bioactive compounds such as porphyran and fucoidan, respectively (33–35). Lower blood cholesterol and triglyceride levels mean a lower risk of atherosclerosis. According to an early study, higher consumption of legumes was substantially linked to a reduced risk of CHD (36). Beans are an excellent source of providing fiber, protein and phytosterols, all of which have been shown to lower LDL-C (37–39). In addition, a number of bioactive peptides derived from soy protein have been validated in various models for their lipid-lowering, angiotensin-converting enzyme-inhibiting, and antioxidant effects (40). Consuming fish was linked to lower rates of CHD morbidity and death, according to a study by Zhang et al. (41). Omega-3 fatty acids, which have been shown to be highly effective in reducing blood triglyceride levels, preventing thrombosis, functioning as an anti-inflammatory, and preventing cardiac arrhythmias, are primarily responsible for fish's capacity to prevent CHD (42–44). Li et al. (45) found a link between consuming 100 g/d of green leafy vegetables and a lower risk of CHD after combining 24 meta-analyses. On the one hand, the soluble dietary fiber in vegetables binds with bile acids, thereby reducing the reabsorption of cholesterol, thus lowering cholesterol levels (46). On the other hand, the antioxidant substances in vegetables can scavenge free radicals and protect vascular endothelial cells (47–49). Although belonging to the same snack category, relevant studies have shown that intake of whole grain bread and chocolate was significantly linked to a lower risk of CHD (50, 51), whereas high intake of white bread, biscuits, cream cakes and fried foods significantly increased the risk of CHD (52, 53). It is reasonable to speculate that some of the adverse cardiovascular effects of snacking are canceled out by other beneficial ingredients. There is ongoing debate on the relationship between eating eggs and the risk of CHD. Even increasing the frequency of egg consumption from less than one egg per month to more than seven eggs per week did not raise the risk of CHD overall, according to a study that combined seven US prospective cohorts (54). Another meta-analysis of prospective dose-response trials came to the same conclusion (55). However, more frequent egg consumption was linked to a lower incidence of ischemic heart disease, according to a cohort research that included over 500,000 Chinese individuals (56). In conclusion, there was no correlation found between decreased egg intake and an increased risk of CHD.

Our findings indicated an increased risk of dyslipidaemia among individuals who followed dietary pattern 3. Similarly, a Brazilian cross-sectional study made clear that girls' high triglyceride levels were linked to dietary patterns high in dairy products, sugary sodas, and sweets (57). A prospective cohort study of adults in Harbin, China, suggested that adherence to a snacking dietary pattern dominated by biscuits, fried crisps, liquid beverages, candies, and ice cream raised the risk of hypercholesterolemia and hypertriglyceridemia (58). Moreover, the results of a study conducted by the National Health and Nutrition Examination Survey in Japan revealed a positive correlation between a dietary pattern characterized by the consumption of bread and dairy products and elevated LDL-C levels, as well as increased total cholesterol levels, in female subjects (59). A cross-sectional study encompassing dietary data from 11,404 Korean women revealed that dietary patterns characterized by elevated consumption of red meat, milk and dairy products, and bread and snacks exhibited a positive correlation with increased total cholesterol levels (60). There are several possible explanations for this association. Firstly, beverages usually contain high levels of fructose, which can induce dyslipidaemia by providing substrates for fatty acid and triglyceride synthesis as well as activating key transcription factors to increase lipogenesis (61, 62). The intake of sugary drinks is positively correlated with triglycerides and LDL-C and negatively correlated with high density lipoprotein cholesterol, which has been confirmed by a large number of scholars (63–66). Second, a lot of processed meals, such baked products and fried foods, are frequently heavy in trans fatty acids and saturated fats, which raise LDL-C levels (67–70). At the same time, snacks are generally high in calories, and excessive intake of snacks can easily lead to obesity, which is one of the important triggers of high blood cholesterol (71). However, the effect of dairy products on blood lipids remains controversial. Low-fat dairy products, which are high in saturated fatty acids, are advised to be substituted for full-fat dairy products according to traditional dietary standards. However, recent studies have demonstrated that full-fat dairy products do not have a negative effect on the lipid profile (72–74). The specific mechanism of dairy products' role in this needs to be explained by further research.

In this study, dietary pattern 1 was not associated with the risk of cardiovascular disease (CVD). This might be due to the fact that the protective components counteracted the effects of potential risk factors. For instance, grains were classified into whole grains and refined grains, and meats included poultry and red meat, but they had different effects on CVD. A meta-analysis that included 43 observational studies indicated that red meat consumption was positively correlated with CVD (75). Another systematic review of prospective cohort studies pointed out that replacing red meat or processed meat with poultry was negatively correlated with the risk of CVD, CHD and stroke (76). The 2020 Dietary Guidelines Advisory Committee concluded that one of the characteristics of dietary patterns associated with reducing the risk of CVD is a higher intake of whole grains and a lower intake of red meat and processed meat, as well as refined grains (77). In addition, the lack of association might be due to the fact that our analysis did not take into account some potential confounding factors, such as alcohol consumption and smoking status.

This study boasts several advantages. Firstly, dietary patterns derived through factor analysis offer a more realistic portrayal of actual eating habits compared to priori patterns. Secondly, data collection was handled by highly skilled professionals who ensured quality control throughout the process, guaranteeing information accuracy. Lastly, to address key potential confounders, three statistical models were employed for this investigation. However, this study has certain limitations. Initially, the causal relationship between common chronic diseases and dietary habits was difficult to as-certain due to the inherent limitations of cross-sectional studies. Further validation of our results is required through either prospective cohort studies or randomized con-trolled trials to ensure robustness. Secondly, the use of the FFQ to assess dietary intake over the year may be impacted by recall bias. Meanwhile, the categorization of food groups, the retention of factor numbers and the definition of dietary patterns were subjectively determined by the researchers. Therefore, the current dietary patterns may not reflect the full reality of the study population. Third, due to differences in ethnicity, culture, and dietary habits, different populations have different dietary pat-terns, and all the data in this study came from Shandong, China, which would limit the generalisability of the results. Fourth, many potential residual confounding factors were not included in our model, such as educational level, income level, smoking, drinking status, etc., which would affect our results. Fifth, the age range of the population in this study was relatively wide. Although the age factor had been adjusted, it may mask the heterogeneous influence of dietary patterns among different age groups.

## 5 Conclusions

In summary, the current study found that adult residents of Shandong Province, China, had different dietary patterns and that these patterns were related to cardiovascular health. In particular, it was discovered that dietary pattern 2, which was defined by reduced consumption of eggs and higher consumption of edible fungi and algae, legumes, snacks, aquatic products, and vegetables, had a preventive impact against CHD. On the other hand, a higher risk of dyslipidemia was associated with dietary pattern 3, which was characterized by a high intake of dairy products, beverages, and snacks. These findings highlight how important dietary practices are in preventing chronic illnesses and offer insightful information for the creation of nutrition-related policy. Further prospective studies in diverse populations are warranted to validate these findings and enhance our understanding of the broader implications of dietary patterns on health outcomes.

## Data Availability

The data analyzed in this study is subject to the following licenses/restrictions: The data are not publicly available due to privacy and legal reason. Requests to access these datasets should be directed to Guangjian Wu, niuniu10288888@163.com.
