# Supplementary material for: The association of dietary pattern with the risk of common chronic diseases in Shandong Province, China: a cross-sectional study
Source: Front Public Health. 2025 Aug 26;13:1629284. doi: 10.3389/fpubh.2025.1629284 (PMC12417157; doi:10.3389/fpubh.2025.1629284)
Supplement: Supplementary file 1 [file Supplementary_file_1.docx]

Table S1 Details of food group categorization.

| Food group | Food species |
| --- | --- |
| Grains and tubers | Rice and its products, wheat flour and products, fried pasta products, coarse grains, tubers (potato/taro/sweet potato, etc.), miscellaneous beans (green/red/flower beans, etc.) |
| Legumes | Soybeans, soya milk, tofu, other soya products, etc. |
| Edible fungi and algae | Edible fungi, mushrooms, nori, kelp |
| Fruits | Fresh fruits |
| Vegetables | Fresh and dried vegetables |
| Nuts | Peanuts, pistachios, hazelnuts, etc. |
| Meat | Pork, beef, lamb, poultry, animal offal, meat products, etc. |
| Eggs | Fresh eggs, salted duck eggs, preserved eggs |
| Aquatic products | Marine fish, freshwater fish, shrimp, crab, etc. |
| Dairy | Liquid milk, milk powder, yoghurt, cheese, ice cream |
| Snacks | Bread, biscuits, chocolate, candies, puffed food, dried fruits, etc. |
| Beverages | Sugary drinks, juice, coffee, tea, etc. |


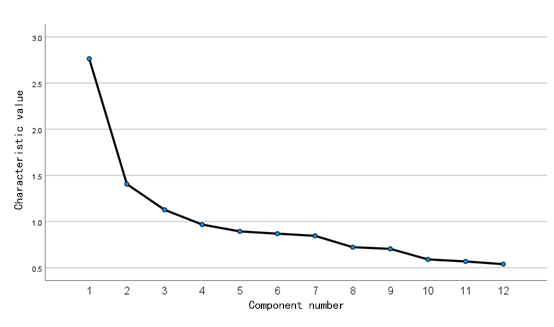


Figure S1 The scree plot.
